# The Monitoring Paradox: Frontal Electroencephalography Reveals Profound Cortical Depression Despite Cardiovascular Stability in Dogs Undergoing Tibial Plateau Leveling Osteotomy with Isoflurane Anesthesia and Multimodal Analgesic Protocols

**DOI:** 10.3390/vetsci13090990

**Published:** 2026-09-19

**Authors:** Jeff C. Ko, Alejandro Vargas Araya, Carla Murillo, Hsin-Yi Weng, Ann B. Weil, Tomohito Inoue

**Affiliations:** 1 Department of Veterinary Clinical Sciences, College of Veterinary Medicine, Purdue University, West Lafayette, IN 47907, USA; avargasa@purdue.edu (A.V.A.); murilloc@purdue.edu (C.M.); aweil@purdue.edu (A.B.W.); tinoue@purdue.edu (T.I.); 2 Department of Comparative Pathobiology, College of Veterinary Medicine, Purdue University, West Lafayette, IN 47907, USA; weng9@purdue.edu

**Keywords:** frontal electroencephalography, parasympathetic tone activity, depth of anesthesia, veterinary anesthesia, fentanyl, epidural morphine, regional blocks, dogs, TPLO, anesthetic safety, autonomic stability

## Abstract

Conventional monitoring relies on autonomic reflexes but fails to directly measure cortical hypnosis, creating a critical information gap regarding true brain state. The objective of this article was to evaluate the cardiorespiratory and cortical responses using frontal EEG and parasympathetic tone activity (PTA) in dogs under isoflurane anesthesia and distinct analgesic protocols. Twenty-one adult dogs (*n* = 7 per group) undergoing tibial plateau leveling osteotomies were assessed at 12 procedural time points using heart rate, blood pressure, Patient State Index (PSI), suppression ratio (SR), and PTA. Treatments included isoflurane maintenance with either intravenous fentanyl, epidural morphine, or regional local blocks. Despite clinically acceptable cardiorespiratory indices, significant treatment-by-time interactions occurred for PSI (p=0.003) and SR (p=0.050). The fentanyl group experienced the deepest cortical suppression and highest Double-Low state incidence compared to epidural morphine (p=0.024). Rapid emergence from these suppressed states yielded dysphoric recoveries in 9 of 21 dogs. In conclusion, evaluating cardiorespiratory and cortical responses demonstrates that standard autonomic monitoring can mask severe intraoperative cortical depression; integrating EEG and PTA offers a valuable approach to optimizing neurophysiological stability.

## Simple Summary

Veterinarians routinely monitor anesthetized dogs using heart rate and blood pressure, but these vital signs may not capture everything happening inside the brain. This study investigated whether frontal electroencephalography (EEG) and heart rate variability monitoring, assessing autonomic balance, could reveal hidden neurophysiological changes in 21 dogs undergoing tibial plateau leveling osteotomies across three analgesic protocols: fentanyl infusion, epidural morphine, and regional local blocks. We discovered a critical monitoring gap. While cardiovascular parameters remained clinically acceptable, EEG revealed frequent, profound cortical suppression with low Patient State Index (≤25) values and high burst suppression ratios (≥20%). This severe brain depression was invisible to standard monitors, especially during fentanyl administration. Furthermore, dogs waking rapidly from these depressive states frequently experienced dysphoria. Despite reasonable median emergence times of 16.0 to 19.0 min, 9 of 21 dogs exhibited rough recoveries, requiring rescue sedation, with scores ranging from 4 to 5 on a scale where 1 is excellent and 5 is very poor. Ultimately, relying solely on cardiovascular monitoring provides an incomplete picture. Integrating frontal EEG monitoring provides a more objective measure of anesthetic depth, thereby optimizing neurophysiological stability and likely improving postoperative outcomes.

## 1. Introduction

General anesthesia is a drug-induced, reversible state defined by unconsciousness, amnesia, immobility, and reduced autonomic responses to noxious stimulation (antinociception) [1,2,3]. Although anesthetic agents act broadly throughout the central nervous system, the cerebral cortex is far more sensitive to anesthetic-induced depression than subcortical structures such as the brainstem and thalamus [4,5,6]. Clinical and experimental evidence shows that anesthetics primarily disrupt cortical communication required for conscious awareness while still allowing nociceptive and sensory signals to reach the brain, potentially triggering activation of the autonomic nervous system if nociception is unmitigated [6,7,8]. Consequently, modern anesthetic practice treats hypnosis and antinociception as distinct therapeutic targets, each requiring individualized drug titration to modulate cortical and subcortical networks during surgery [3,4,5].

Loss of consciousness arises largely from fragmentation of cortical connectivity [5]. Contemporary systems neuroscience further clarifies that anesthesia operates through two interrelated dimensions: degradation of the content of consciousness through top-down frontoparietal networks and suppression of the level of consciousness through bottom-up arousal pathways [6,8,9]. Despite this dual framework, veterinary monitoring continues to rely heavily on autonomic reflexes mediated by the brainstem, including heart rate, respiratory rate, and arterial pressure [1,10,11]. These variables serve as indirect indicators of antinociception rather than direct measures of cortical hypnosis [1,11]. The 2025 American College of Veterinary Anesthesia and Analgesia (ACVAA) monitoring guidelines emphasize physical signs such as muscle tone, eye position, reflex responses, and end-tidal inhalant concentration [12]. These parameters primarily reflect spinal and brainstem physiology [4,10]. Because subcortical structures are evolutionarily resistant to anesthetic depression, they may remain stable even when cortical activity is profoundly suppressed [4,6,13]. This creates a monitoring paradox in which subcortical homeostasis appears preserved while the cortex, the primary pharmacologic target, undergoes deep and often unrecognized depression [6,11].

Human anesthesia has addressed this gap by incorporating processed electroencephalography (EEG) and Parasympathetic Tone Activity (PTA) to directly quantify cortical and autonomic dynamics [1,2,13,14,15]. EEG-derived indices such as the Bispectral Index (BIS), entropy, and the Patient State Index (PSI), together with raw EEG and spectrograms, provide real-time evaluations of cortical state and help prevent awareness and excessive anesthetic exposure [1,2,6,16]. PTA complements these measures by analyzing heart rate variability, which serves as an indirect assessment of autonomic nervous system activity and quantifies beat-to-beat autonomic responsiveness to nociception [14,15,17]. Collectively, these modalities offer a multimodal characterization of brain state that traditional hemodynamic monitoring cannot capture [7,13].

Distinct EEG signatures produced by different anesthetic classes further illustrate the mechanisms of unconsciousness [3,7]. Volatile agents, propofol, and opioids generate characteristic patterns that reflect their effects on cortical connectivity, whereas dissociatives such as ketamine may increase cortical activity despite producing unconsciousness [3,6,7]. Profound cortical suppression, including burst suppression, is associated with delayed emergence and postoperative cognitive dysfunction in humans [11,18,19,20,21]. These insights highlight the value of direct cortical monitoring, especially in multimodal protocols where synergistic drug interactions can produce deep cortical depression despite stable hemodynamic variables [6,7,22].

In canine orthopedic procedures such as Tibial Plateau Leveling Osteotomy (TPLO), multimodal protocols commonly combine alpha-2 agonists, dissociatives, potent opioids, and regional anesthesia. Systemic opioids and sedatives substantially reduce the inhalant concentration required for immobility, increasing the risk of a Triple-Low state characterized by hypotension, low inhalant concentration, and profound cortical suppression [3,11,23,24,25,26]. In humans, Triple-Low states are associated with delayed extubation, emergence delirium, and impaired recovery trajectories [11,18,19,20,27]. Clinically, dogs undergoing these procedures often experience prolonged or difficult recoveries that require antagonism of alpha-2 agonists or opioids. Combined with the known sensitivity of cortical networks to anesthetic-induced disconnection, these observations suggest that important aspects of anesthetic depth may go unrecognized when relying solely on hemodynamic variables [1,6,7].

While conventional veterinary anesthetic monitoring relies almost exclusively on subcortically mediated cardiorespiratory reflexes to gauge anesthetic depth and nociceptive responsiveness, these autonomic indicators do not measure cerebral cortical hypnosis. Consequently, profound drug-induced cortical depression can occur unnoticed in patients who appear hemodynamically stable, predisposing them to post-anesthetic neurobehavioral complications [4]. Although processed frontal EEG and PTA monitor cortical state and autonomic tone, respectively, their concurrent utility across differing analgesic regimens in clinical veterinary patients remains uncharacterized [13,15,16].

Therefore, the objective of this article was to evaluate the cardiorespiratory and cortical responses using frontal EEG and PTA in dogs under isoflurane anesthesia and three distinct analgesic regimens—intravenous fentanyl constant rate infusion (FC), lumbosacral epidural morphine (ME), and ultrasound-guided peripheral nerve blocks (RB)—during tibial plateau leveling osteotomy (TPLO). We hypothesized that: (1) frontal EEG would identify profound cortical suppression and “Double-Low” states that are undetectable by conventional cardiovascular monitoring; and (2) dogs receiving systemic opioid infusions (FC) would exhibit significantly deeper intraoperative cortical depression and a higher incidence of dysphoric recoveries than dogs managed with neuraxial (ME) or locoregional (RB) analgesia.

## 2. Materials and Methods

### 2.1. Animals and Study Design

Twenty-one client-owned dogs presenting consecutively to the Purdue University Veterinary Hospital for unilateral tibial plateau leveling osteotomy (TPLO) between August 2024 and July 2025 were prospectively screened and enrolled in this randomized clinical trial. Written informed consent was obtained from all owners prior to enrollment. The study protocol was approved by the Purdue University Institutional Animal Care and Use Committee (PACUC #0724002513PA277) and conducted in accordance with institutional ethical guidelines and regulations. All dogs were classified as American Society of Anesthesiologists Physical Status II based on clinical history, physical examination, complete blood count, and serum biochemistry. Dogs were excluded if they had pre-existing neurological disease, cardiac arrhythmias, skin allergy above the surgical site, or had received analgesic or sedative medications within 24 h of surgery.

An a priori sample size calculation was performed to determine the number of dogs required per treatment group. Clinically meaningful differences were defined using thresholds that reflect substantive neurophysiologic and hemodynamic change, recognizing that absolute baseline MAP and heart rate vary considerably among medium-sized dogs. For EEG-derived variables, a 20-point change in PSI and a 20% change in SR were selected because they represent transitions across the manufacturer-defined surgical zone (PSI 25–50) and indicate a meaningful increase in cortical depression (SR ≥ 20%). For hemodynamic variables, ≥20% changes in MAP and heart rate were considered more physiologically relevant than fixed absolute cutoffs, as percentage-based criteria better capture clinically important deviations from each individual dog’s resting state; a 20% MAP reduction reliably approaches perfusion-risk territory, and a 20% HR change reflects a meaningful autonomic response to anesthetic depth or cardiovascular compromise. Using these predefined effect sizes, the power analysis was conducted with a two-sided α of 0.05 (type I error rate) and 80% power (1 − β = 0.80) to detect differences between treatment groups. This analysis determined that a minimum of *n* = 7 dogs per group was required to detect a statistically significant difference in the primary EEG indices.

### 2.2. Anesthetic and Analgesic Protocols

Dogs were randomly assigned to one of three analgesic treatment groups (*n* = 7 per group) using a computer-generated randomization sequence prior to the start of the study. The three treatment groups consisted of an intravenous fentanyl constant rate infusion (FC), a lumbosacral epidural morphine injection (ME), and an ultrasound-guided regional block consisting of sciatic and saphenous nerve blocks (RB). The FC group received an intravenous fentanyl (Fentanyl 0.05 mg/mL Injectable, Hospira, Lake Forest, IL, USA) loading dose of 5 µg/kg followed by a constant rate infusion of 5 µg/kg/h. The ME group received a lumbosacral epidural injection of preservative free morphine (Morphine Sulfate 1 mg/mL, Hospira, Lake Forest, IL, USA) at 0.1 mg/kg, diluted 1:1 with 0.9% sodium chloride (Hospira, Lake Forest, IL, USA) to a total volume of 0.2 mL/kg, administered using a 22 gauge spinal needle (BD, Franklin Lakes, NJ, USA or RapID™, Irving, TX, USA). The RB group received ultrasound-guided sciatic and saphenous nerve blocks using 0.5% bupivacaine (Hospira, Lake Forest, IL, USA) at 0.1 mL/kg per site, performed with a Mindray TE7 Max Advance ultrasound unit (Mahwah, NJ, USA) and a 21 G × 4″ echogenic needle (Medline^®^, Northfield, IL, USA). All epidural injections and ultrasound-guided regional nerve blocks were performed by a single board-certified anesthesiologist to maintain procedural uniformity across cases. All analgesic interventions (fentanyl bolus/CRI initiation, epidural injection, and both regional nerve blocks) were completed within 8 min of anesthetic induction.

All dogs received a standardized anesthetic and perioperative regimen. Premedication consisted of intramuscular dexmedetomidine (5 µg/kg, Dexdomitor 0.5 mg/mL, Zoetis, Kalamazoo, MI, USA) and hydromorphone (0.1 mg/kg, Hydromorphone 2 mg/mL, Hikma, Berkeley Heights, NJ, USA). After approximately fifteen to twenty minutes, a cephalic vein was catheterized (IV catheter, Terumo™, Somerset, NJ, USA), and anesthesia was induced with propofol and ketamine. The induction was performed by administering 1 mg/kg propofol (Propoflo 10 mg/mL, Zoetis, Kalamazoo, MI, USA) followed immediately by ketamine (2 mg/kg, Ketamine 100 mg/mL, Dechra, Overland Park, KS, USA), then titrating the additional 1 mg/kg propofol to effect to allow endotracheal intubation. Balanced electrolyte fluids (pHyLyte, Dechra, Overland Park, KS, USA) were administered at 5 mL/kg/h throughout anesthesia. Standard pharmacological dosing guidelines were followed for all analgesic and anesthetic interventions, using the dosages commonly employed at the Purdue University Veterinary Teaching Hospital and consistent with recommendations in *Small Animal Anesthesia and Pain Management: A Color Handbook* [28].

Anesthesia was maintained with isoflurane (Fluriso, Vet One, Austin, TX, USA) in 100% oxygen using an anesthesia machine and ventilator (Mindray WATO Ex 35, Mahwah, NJ, USA). All dogs were mechanically ventilated in pressure-controlled mode to maintain end tidal carbon dioxide (Et-CO_2_) between 35 and 45 mmHg. The end tidal isoflurane (Et-Iso) concentration was initially set between 1.0 and 1.3% and subsequently adjusted based on conventional clinical indicators of anesthetic depth, including eye-globe position, corneal and palpebral reflexes, jaw tone, and hemodynamic stability. EEG and PTA were recorded throughout anesthesia for observational purposes only and did not influence anesthetic management. At the end of surgery, isoflurane was discontinued, and the time to extubation was recorded. Extubation was defined as the return of the swallowing reflex followed by removal of the endotracheal tube.

### 2.3. Cardiorespiratory, Electroencephalographic, and Parasympathetic Tone Activity (PTA) Monitoring

A multiparameter monitor (Mindray Multiparameter Monitor, Mahwah, NJ, USA) was used for all physiologic monitoring. Heart rate and cardiac rhythm were assessed using a lead II ECG. Pulse rate (HR) was obtained from pulse oximetry, and direct arterial blood pressure, including systolic blood pressure (SBP), mean blood pressure (MBP), and diastolic blood pressure (DBP), was measured via an arterial catheter (Terumo™, Somerset, NJ, USA) placed in the dorsal pedal artery. Et-CO2 and Et-Iso concentrations were measured using a side-stream capnograph with anesthetic agent monitoring capability (Mindray Side-stream Capnograph, Mahwah, NJ, USA). Body temperature was monitored with an esophageal probe during surgery and transitioned to a rectal probe after extubation. All instrumentation was completed within 10 min after induction, and physiologic variables were recorded every 5 min.

Surrogate indicators of anesthetic depth were also assessed, including eye globe position, corneal and palpebral reflexes, jaw tone, and front-limb muscle relaxation. Frontal EEG was recorded using a modified SedLine^®^ system adapted for canine skull morphology [29,30]. Adult SedLine^®^ adhesive electrodes were converted to subdermal needle electrodes (Ambu, Columbia, MD, USA) to improve contact and avoid clipping. Before each procedure, electrodes were tested and verified by the SedLine Root^®^ monitor, which requires impedance values between 0.0 and 65.1 kΩ. Electrodes were positioned analogously to the human 10–20 system following the established canine montage: R1 at Fp2, R2 between F4 and F8, L1 at Fp1, and L2 between F3 and F7. The ground (CB) electrode was placed along the mid-sagittal line, and the reference (CT) electrode was placed cranially along the same line. All EEG-derived indices, including the Patient State Index (PSI), Suppression Ratio (SR), 95% Left and Right Spectral Edge Frequency (SEF-L and SEF-R), and Density Spectral Array (DSA) parameters, were continuously recorded. High-resolution EEG data were exported as CSV files at 2 s intervals and downloaded from the Sedline monitor (Masimo Corporation, Irvine, CA, USA).

PTA monitoring was performed using the MDoloris system (Loos, France), which analyzes beat-to-beat R R interval variability. The PTA device uses a three-lead ECG configuration with alligator clips, integrated into the existing lead II setup. A green icon confirmed adequate signal acquisition. Only the mean PTA (mPTA) was used for analysis. Immediate PTA was not considered because mPTA provides a more stable reflection of autonomic balance over the 5 min clinical recording intervals. The mPTA is calculated from the most recent 176 R-R intervals, representing a 64 s moving window. On the PTA scale (0–100), values between 50 and 70 indicate an optimal analgesia–nociception balance according to the manufacturer’s clinical guidelines [14,15], whereas values below 50 reflect sympathetic predominance.

### 2.4. Recovery Assessment and Postoperative Analgesic Protocol

Recovery evaluation began at the termination of isoflurane and continued through emergence and extubation. Recovery quality was assessed using a simple descriptive scale: 1 (excellent), 2 (good), 3 (acceptable), 4 (poor), and 5 (very poor). Emergence was classified as “smooth” for scores of 1, 2, or 3, and “rough” for scores of 4 or 5. After extubation, postoperative pain was assessed at 1, 2, 4, 8, 12 h, and before discharge (<12 h) using the Glasgow Composite Measure Pain Scale–Short Form (CMPS-SF) [31]. Rescue analgesia consisted of hydromorphone (0.05 mg/kg IV; Hydromorphone 2 mg/mL, Hikma, Berkeley Heights, NJ, USA) and was administered if the CMPS-SF score exceeded 6/24 (or 5/20 for non-ambulatory dogs). Both the frequency of rescue analgesia and total postoperative opioid consumption were recorded.

### 2.5. Data Processing and Statistical Analysis

#### 2.5.1. Data Integration and Time-Point Standardization

To account for variation in surgical duration, all physiologic, hemodynamic, EEG, and nociception-related data were aligned to a standardized procedural timeline. Processed EEG indices were sampled every 2 s, while hemodynamic variables and mPTA were recorded at 5 min intervals. The anesthetic and surgical period was divided into twelve clinically defined intervals that reflect consistent procedural milestones across all dogs. Time point 1 (T1) corresponded to premedication, followed by Time point 2 (T2) for induction and intubation. Draping and surgical readiness were completed at Time point 3 (T3), and arthroscopy was performed at Time point 4 (T4). The first skin incision was designated Time point 5 (T5), while Time point 6 (T6) encompassed muscle and fascia dissection, as well as periosteal elevation. Bone drilling, bone sawing, and osteotomy were grouped within Time point 7 (T7), and pin or implant placement occurred during Time point 8 (T8). Suturing and closure of the muscle and skin were represented at Time point 9 (T9), with the end of the procedure captured at Time point 10 (T10). Termination of inhalant anesthesia occurred at Time point 11 (T11), and extubation occurred at Time point 12 (T12). Values within each interval were averaged to generate a single representative value for statistical modeling.

#### 2.5.2. Clinical Definitions and Descriptive Analysis of Low States

In contrast to the interval-based averaged values described above, Double-Low and Triple-Low states were identified from the unaveraged, continuously sampled physiologic and anesthetic data (raw data), evaluated across three domains: cortical activity, arterial pressure, and inhalant concentration. The definitions used in this study were adapted from the human perioperative anesthesia literature [9,10,11,12,18], in which concurrent low cortical activity, hypotension, and low volatile anesthetic concentrations have been associated with adverse postoperative outcomes. In our adaptation, low cortical activity was defined by a PSI ≤ 25, with SR ≥ 20% indicating a Low Brain State; low arterial pressure was defined as mean blood pressure (MBP) ≤ 60 mmHg; and low inhalant concentration was defined as end tidal isoflurane ≤ 1.02%, corresponding to approximately 0.8 MAC in dogs based on established canine MAC values of 1.28–1.30% [23,24,25,26]. Each raw data point within each time interval was reviewed, and concurrent alignment of any two criteria was classified as a Double-Low, whereas alignment of all three criteria was classified as a Triple Low. These classifications were applied uniformly across all dogs and all recorded intervals.

#### 2.5.3. Statistical Analysis

To determine whether cardiorespiratory parameters, cortical activity, and PTA differed across the distinct analgesic protocols, the hemodynamic, anesthetic, EEG, and PTA variables were analyzed across the twelve clinical time points using linear mixed-effects models. These included HR, SBP, MBP, DBP, Et-Iso, PSI, SR, SEF-L, SEF-R, and mPTA. Analgesic treatment group, time point, and their interaction were initially included as fixed effects, with dog modeled as a random intercept to account for repeated measurements. The treatment-by-time interaction was examined to determine whether the three analgesic techniques differed in their hemodynamic, EEG, and PTA profiles over time. When the interaction was significant, comparisons were made within groups or time points. When the interaction was not significant, the interaction term was not interpreted, and main-effect trends were summarized. Assumptions of the linear mixed-effects models were evaluated using residual diagnostics, including assessments of residual normality, homogeneity of variance, and extreme residual observations. Model singularity was also evaluated.

Contrasts were specified to compare each intra-anesthetic time point (T2 through T11) with the premedication (T1) and extubation (T12) reference points, when data were available. Additional successive time point contrasts were used to evaluate changes across key procedural transitions, particularly from surgical readiness to surgical stimulation and from the end of surgery into recovery. Holm adjustment was applied within each outcome to control the family-wise error rate for these planned temporal contrasts. Contrast estimates with 95% confidence intervals (CI) were reported to describe the magnitude and direction of observed effects. Post hoc comparisons among analgesic treatment groups were performed only after significant model effects were detected, with Tukey adjustment for pairwise differences.

Recovery quality (smooth vs. rough) and rescue analgesia use were evaluated using Fisher’s exact test. Double-Low and Triple-Low events were recorded as binary variables at each clinical time point (T1–T12), and mixed-effects logistic regression was used to compare event occurrence across analgesic techniques and over time. Outcomes with very few events or insufficient between-dog variability were summarized descriptively. Statistical significance for all main effects, interactions, and post hoc comparisons was set a priori at an alpha level of 0.05 (p<0.05). All analyses were performed in R (Version 4.6.0; R Core Team, 2026) and RStudio (Version 2026.07; Posit Team, 2026).

#### 2.5.4. Use of AI-Assisted Editing and Figure Tools

During the preparation of this manuscript, the authors used Google Gemini (Google DeepMind, 2026), Microsoft Copilot (v2026.7), and Grammarly (2026 release) to support targeted language editing and figure refinement. The graphical abstract was generated using natural-language prompting in Google Gemini combined with authentic EEG recordings, followed by iterative adjustments in Microsoft PowerPoint (Office 365, 2026), Corel PaintShop Pro 2022, and Topaz Photo AI (v3.0) to ensure accurate medical annotation and visual clarity. All AI-assisted outputs were independently reviewed, edited, and verified by the authors, who assume full responsibility for the accuracy and integrity of the final work.

## 3. Results

### 3.1. Patient Demographics and Procedural Durations

A total of 21 dogs were enrolled, with no significant differences in demographics (age, weight, sex) observed across the three treatment groups (Table 1). Surgical duration did not differ significantly among groups (mean range: 108–124 min; *p* = 0.612), nor did total anesthesia time (mean range: 231–274 min; *p* = 0.160).

### 3.2. Hemodynamic and Autonomic Trends Across TPLO Time Points

**Figure 1:**
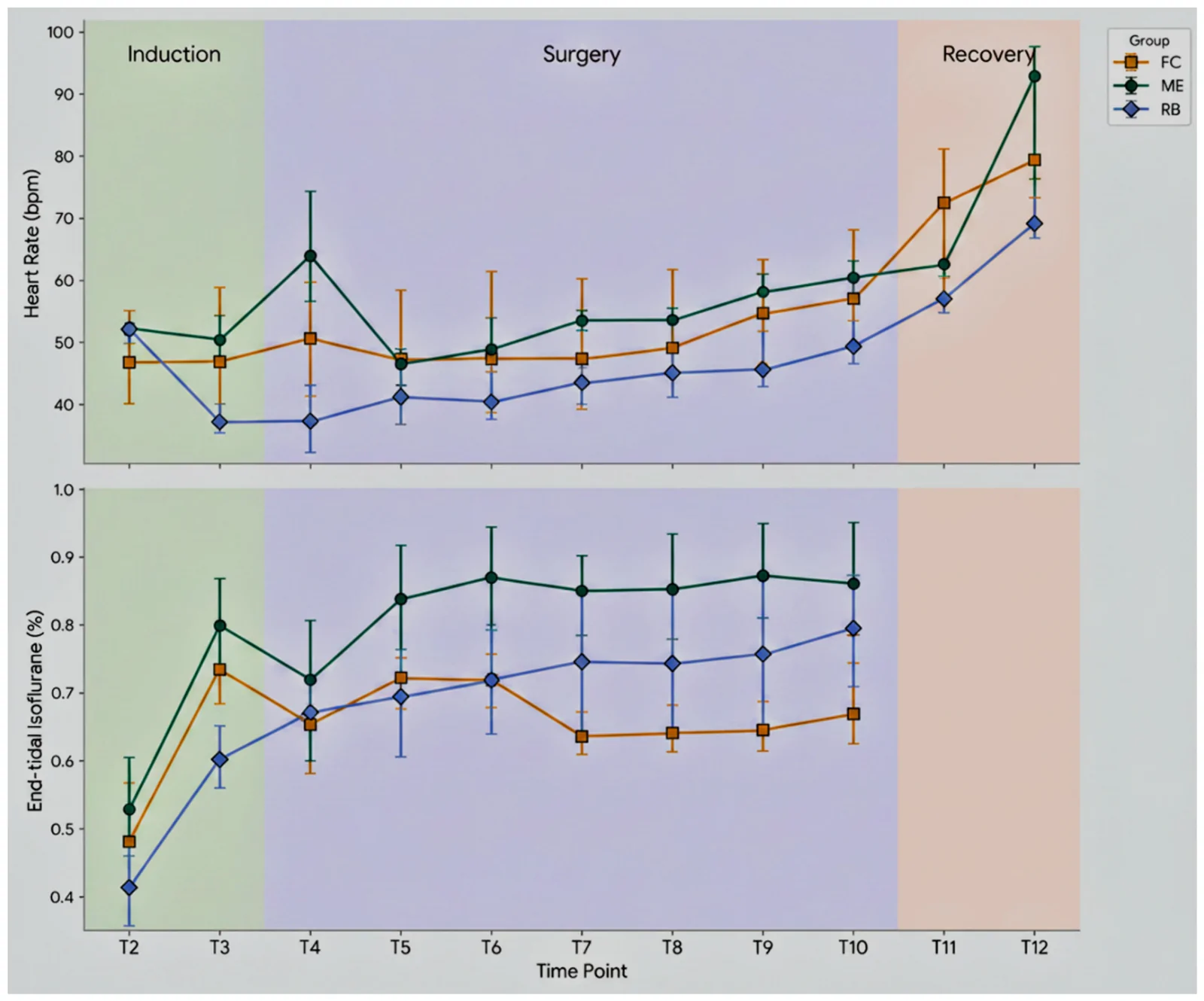
Heart Rate and End-tidal Isoflurane Trends Over the Canine Tibial Plateau Leveling Osteotomy Procedure. Heart rate and end-tidal isoflurane concentrations for the fentanyl infusion (FC; orange squares), epidural morphine (ME; green circles), and regional local block (RB; blue diamonds) treatment groups. Data represent observed group mean ± standard deviation for heart rate (bpm) and end-tidal isoflurane (%) measured at standardized clinical time points (T2–T12). Procedural milestones are shaded as follows: induction and surgical preparation (T2–T3, light green), surgery (T4–T10, light lavender), and recovery (T11–T12, light orange). Isoflurane anesthesia was discontinued following T10, precluding further end-tidal isoflurane measurements.

**Figure 2:**
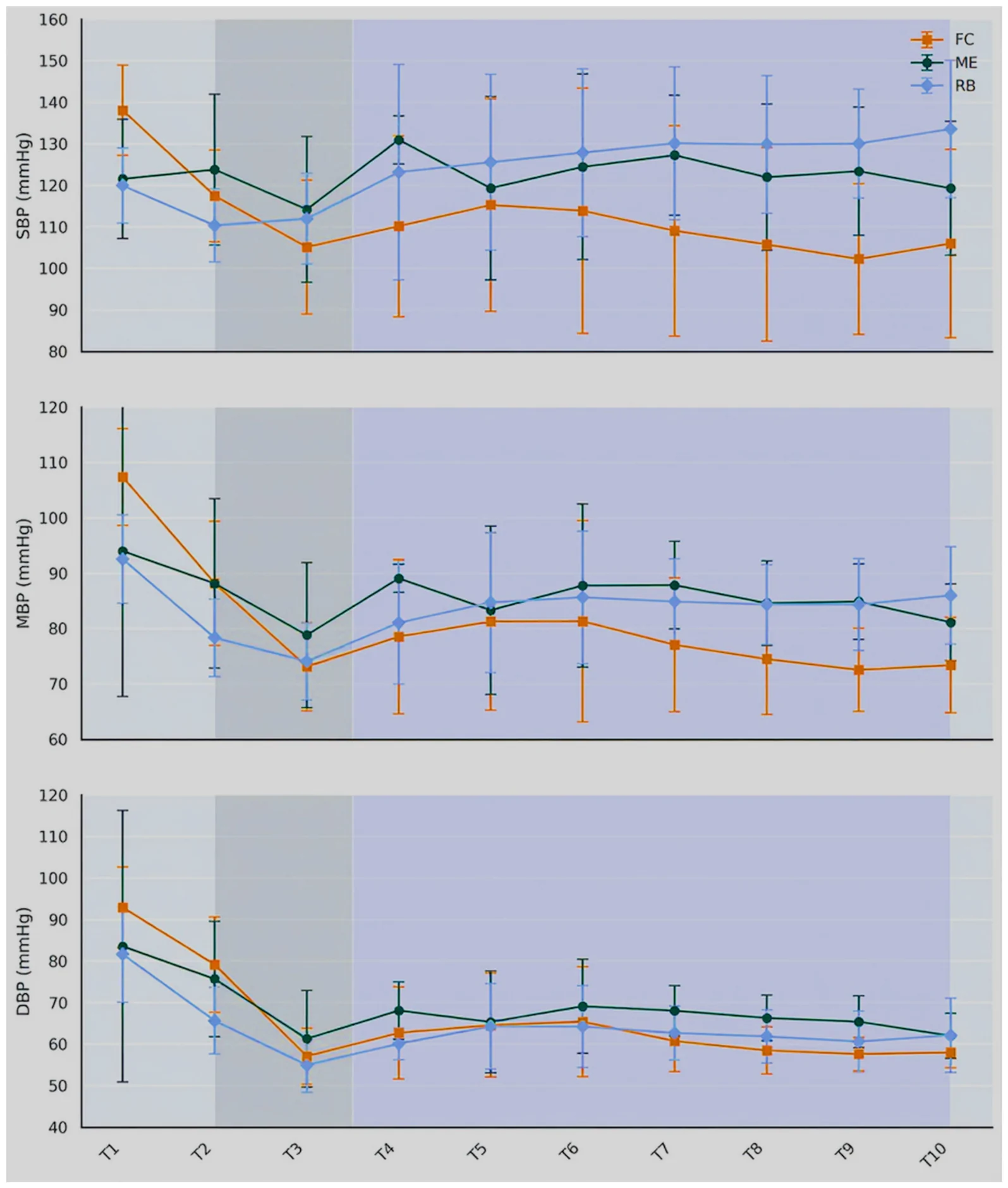
Arterial Blood Pressure Trends During General Anesthesia for Canine Tibial Plateau Leveling Osteotomy. Perioperative arterial blood pressure course for the fentanyl infusion (FC; orange squares), epidural morphine (ME; green circles), and regional local block (RB; blue diamonds) treatment groups. Data represent observed group mean ± standard deviation for systolic, mean, and diastolic blood pressure measured at standardized clinical time points (T2–T10). Procedural milestones are shaded as follows: Premedication (T1–T2, white), induction and surgical preparation (T2–T3, light gray), and surgery (T4–T10, light lavender). The plot illustrates temporal fluctuations throughout the anesthetic procedure, characterized by a transient decline in pressures following the initiation of surgical stimulation at T3.

All physiologic and hemodynamic variables demonstrated significant temporal fluctuations throughout anesthesia and surgery (main effect of time), including HR (*p* < 0.001), MBP (*p* < 0.001), DBP (*p* < 0.001), Et-Iso (*p* < 0.001), mPTA (*p* < 0.001), and SBP (*p* = 0.019). The trend could be observed in Figure 1 and Figure 2. However, the main effect of the treatment group was not significant for any cardiorespiratory parameter, including HR (*p* = 0.352), SBP (*p* = 0.207), MBP (*p* = 0.460), DBP (*p* = 0.581), Et-Iso (*p* = 0.115), or mPTA (*p* = 0.278).

HR, DBP, and mPTA did not demonstrate significant treatment-by-time interactions (*p* = 0.139, *p* = 0.330, and *p* = 0.872, respectively), and their overall temporal patterns were evaluated irrespective of treatment group. For HR (Figure 1), significant temporal changes were detected between extubation (T12) and the termination of isoflurane anesthesia (T11) (*p* < 0.001), as well as between T12 and premedication (T1) (*p* < 0.001). No significant differences in Et-Iso concentration were observed among treatment groups (interaction *p* = 0.442) (Figure 1).

SBP and MBP (Figure 2) demonstrated significant treatment-by-time interactions (*p* = 0.004 and *p* = 0.026, respectively). While these pressures remained within clinically acceptable ranges for all dogs, evaluating the data at individual time points revealed distinct group differences. At the end of surgery (T10), MBP was, on average, 15.0 mmHg (95% CI: −30.1, 0.16; *p* = 0.053) lower in the FC group than in the RB group (Figure 2). SBP was significantly higher in the RB group than in the FC group during joint closure (T9 mean difference: 27.8 mmHg; 95% CI: 3.26, 52.3; *p* = 0.024) and at T10 (mean difference: 31.0 mmHg; 95% CI: 5.74, 56.2; *p* = 0.013) (Figure 2).

### 3.3. Electroencephalographic Trends over the TPLO Procedure

**Figure 3:**
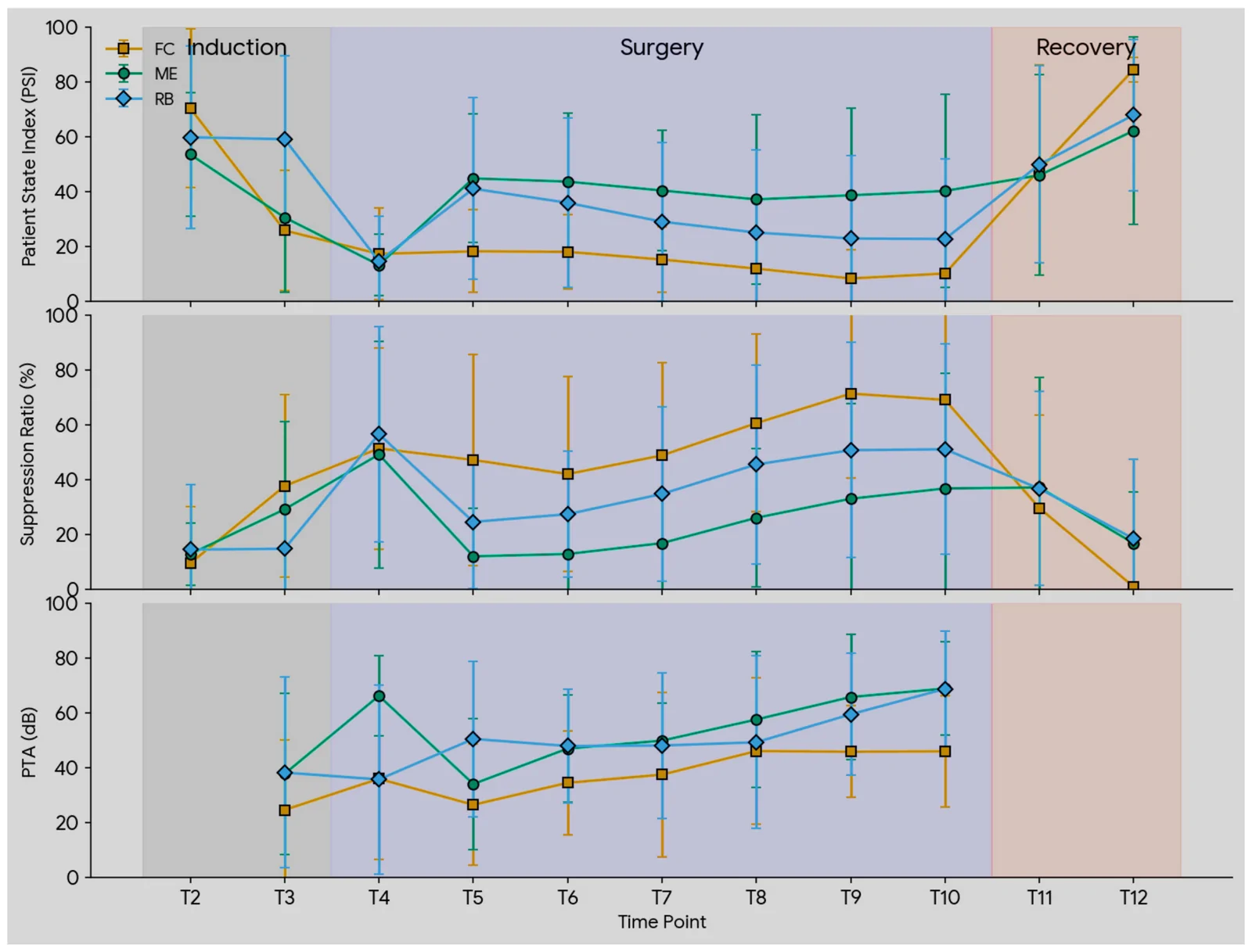
Cortical and Autonomic Monitoring Indices During General Anesthesia for Canine Tibial Plateau Leveling Osteotomy. Patient State Index (PSI, top panel), Suppression Ratio (SR, middle panel), and Parasympathetic Tone Activity (PTA, bottom panel) across procedural time points (T2–T12). Treatment group symbols (FC, ME, RB) and procedural background shading (Induction, Surgery, Recovery) are identical to Figure 1. Data markers represent observed group mean ± standard deviation (SD). All cortical and autonomic indices exhibited significant temporal fluctuations throughout the study, with a decrease in PSI and an increase in SR following the initiation of surgical stimulation.

**Figure 4:**
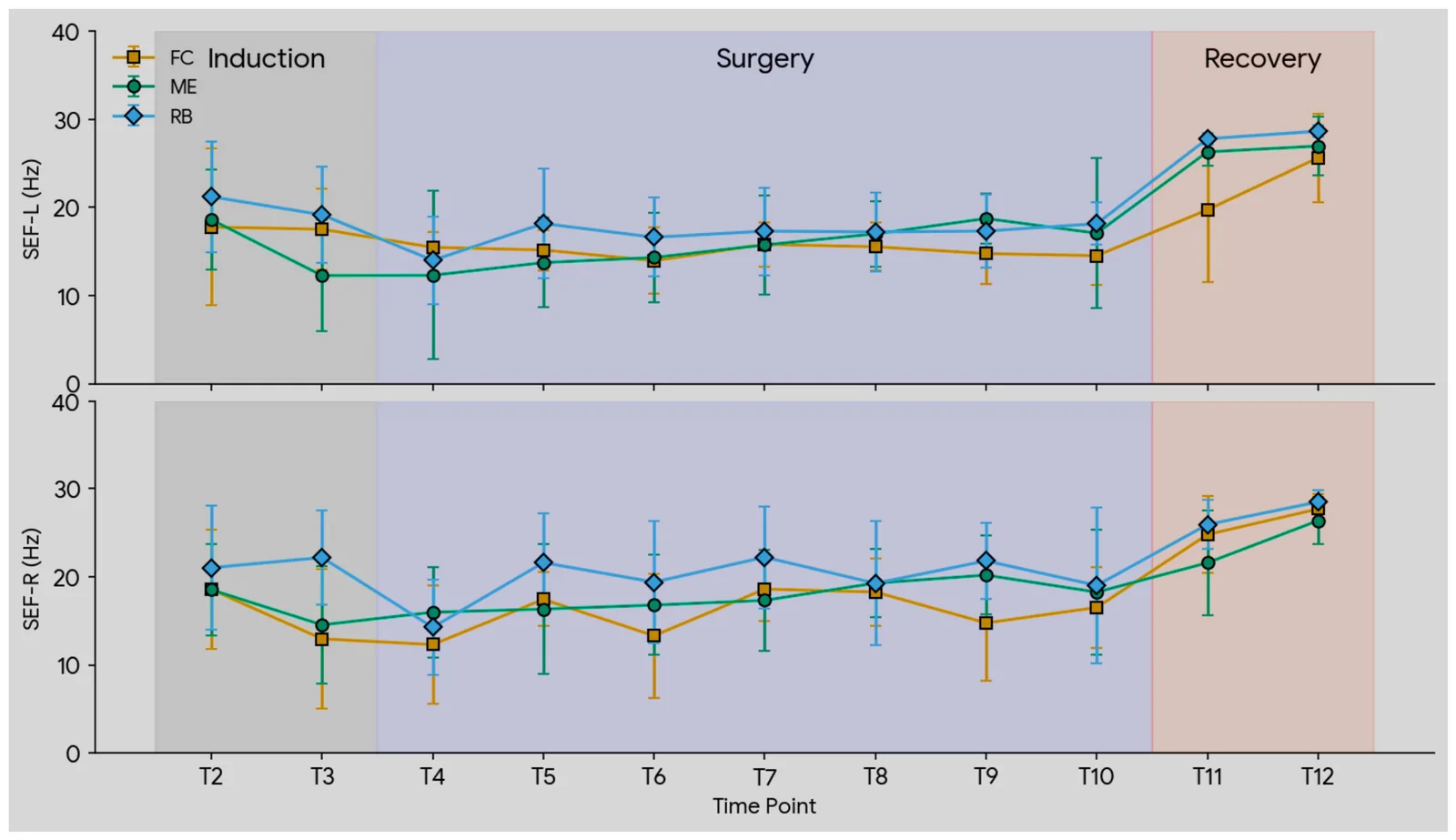
Bilateral 95% Spectral Edge Frequency (SEF) Trends During General Anesthesia for Canine Tibial Plateau Leveling Osteotomy. 95% SEF in the left (SEF-L, top panel) and right (SEF-R, bottom panel) hemispheres across designated clinical time points (T2–T12) in dogs undergoing TPLO surgery. Treatment group symbols and procedural background shading are identical to Figure 1. Data markers represent observed group mean ± standard deviation. All groups demonstrated similar bilateral temporal patterns, with SEF decreasing after anesthetic induction, remaining relatively stable during surgical maintenance, and rising toward pre-induction values during recovery.

Cortical EEG indices (Figure 3 and Figure 4), specifically the PSI and SR (Figure 3), demonstrated significant treatment-by-time interactions (*p* = 0.003 and *p* = 0.050, respectively). Within the FC group, temporal contrasts revealed a significant decline in PSI from T1 to initial surgical readiness (T3) (mean difference: −53.8; 95% CI: −76.2, −31.4; *p* < 0.001). In contrast, this decline was not statistically significant in the RB group at the same transition (mean difference: −23.2; 95% CI: −48.0, 1.63; *p* = 0.334). Within the FC group, significant temporal differences in SR were also observed from T4 to T10 relative to T1 (*p*-values ranging from *p* = 0.043 to *p* < 0.001), with a significant decrease (*p* = 0.004) from the end of surgery (T10) to the termination of isoflurane anesthesia (T11) (Figure 3). No significant within-group temporal variations in SR were observed for the RB or ME groups. Bilateral 95% Spectral Edge Frequencies (SEF-L and SEF-R) (Figure 4) did not demonstrate significant treatment-by-time interactions (*p* = 0.301 and *p* = 0.623, respectively).

### 3.4. Incidence of Double and Triple-Low States

**Figure 5:**
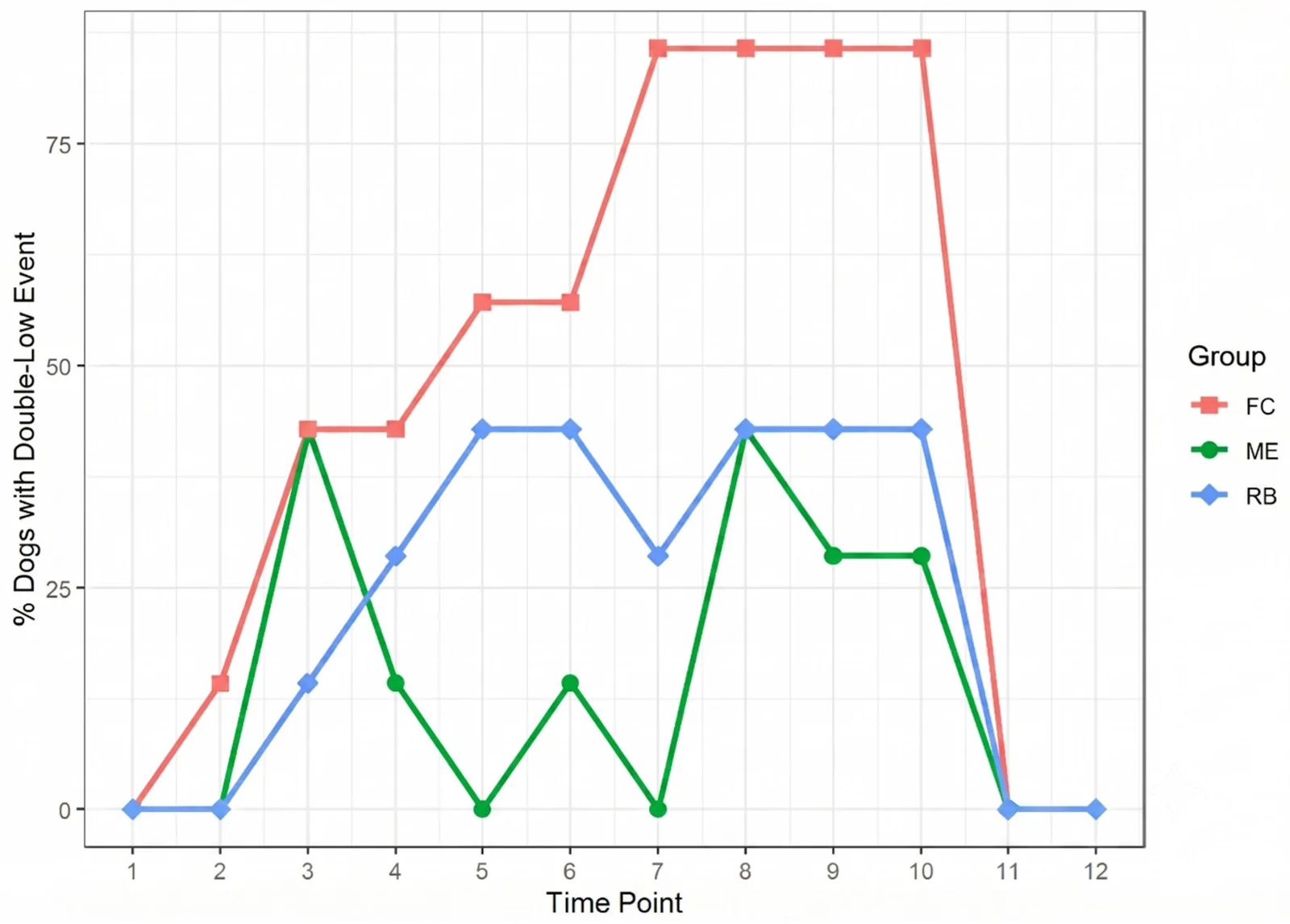
Incidence of Double-Low States Over Time. Percentage of dogs in the fentanyl infusion (FC; orange squares), epidural morphine (ME; dark green circles), and regional local block (RB; light blue diamonds) treatment groups exhibiting a Double-Low state at each clinical time point (T1–T12). Procedural phases are defined as follows: induction and surgical preparation (T2–T3), surgical maintenance (T4–T10), and recovery (T11–T12). The graph displays the temporal progression of these events, showing high-frequency clustering in the FC group throughout the surgical maintenance phase.

A total of 71 Double-Low events (Table 2) were identified, consisting entirely of cortical suppression (Low Brain State) paired with low inhalant requirements. Mixed-effects logistic regression indicated that the incidence of Double-Low states (Figure 5) varied significantly by treatment group (*p* = 0.025). The FC group demonstrated a significantly higher incidence of Double-Low states compared with the ME group (Odds ratio: 7.26; 95% CI: 1.21, 43.5; *p* = 0.024) (Figure 5). No significant differences in Double-Low incidence were detected between the RB and FC groups (*p* = 0.153) or between the RB and ME groups (*p* = 0.339).

### 3.5. Emergence and Immediate Recovery

During the immediate recovery phase, emergence times were comparable among the protocols, with median durations of 19.0 min for the FC group, 19.0 min for the ME group, and 16.0 min for the RB group. At the end of surgery (T10), the FC group showed the greatest cortical suppression, with higher SR and lower PSI than the ME group; however, neither difference was statistically significant (SR mean difference: 32.3; 95% CI: −6.23, 70.7; *p* = 0.117; PSI mean difference: −30.1; 95% CI: −64.1, 3.94; *p* = 0.093). By the termination of inhalant anesthesia (T11), EEG profiles were similar across treatments, with between-group differences in mean SR and PSI no greater than 4.6 and 7.9, respectively. Upon extubation, 5 of 7 dogs in the RB group recovered smoothly. Rough, delirious recoveries were observed in 4/7 dogs in the ME group, 3/7 in the FC group, and 2/7 in the RB group (*p* = 0.854). Rescue sedation or analgesia was administered to 4 dogs in the ME group, 3 in the FC group, and 2 in the RB group during the immediate postoperative period.

### 3.6. Postoperative Pain and 24-Hour Rescue Analgesia

In the 24 h postoperative period, median peak Glasgow Composite Measure Pain Scale–Short Form scores were 3 in the FC group, 2 in the ME group, and 3 in the RB group. Postoperative rescue analgesia interventions were required for 3 of 7 dogs in the FC group (3 total doses) and 2 of 7 dogs in the ME group (4 total doses), while no dogs in the RB group reached the clinical threshold for rescue analgesia (*p* = 0.292).

### 3.7. Perioperative Brain State Progression Through Electroencephalography

**Figure 6:**
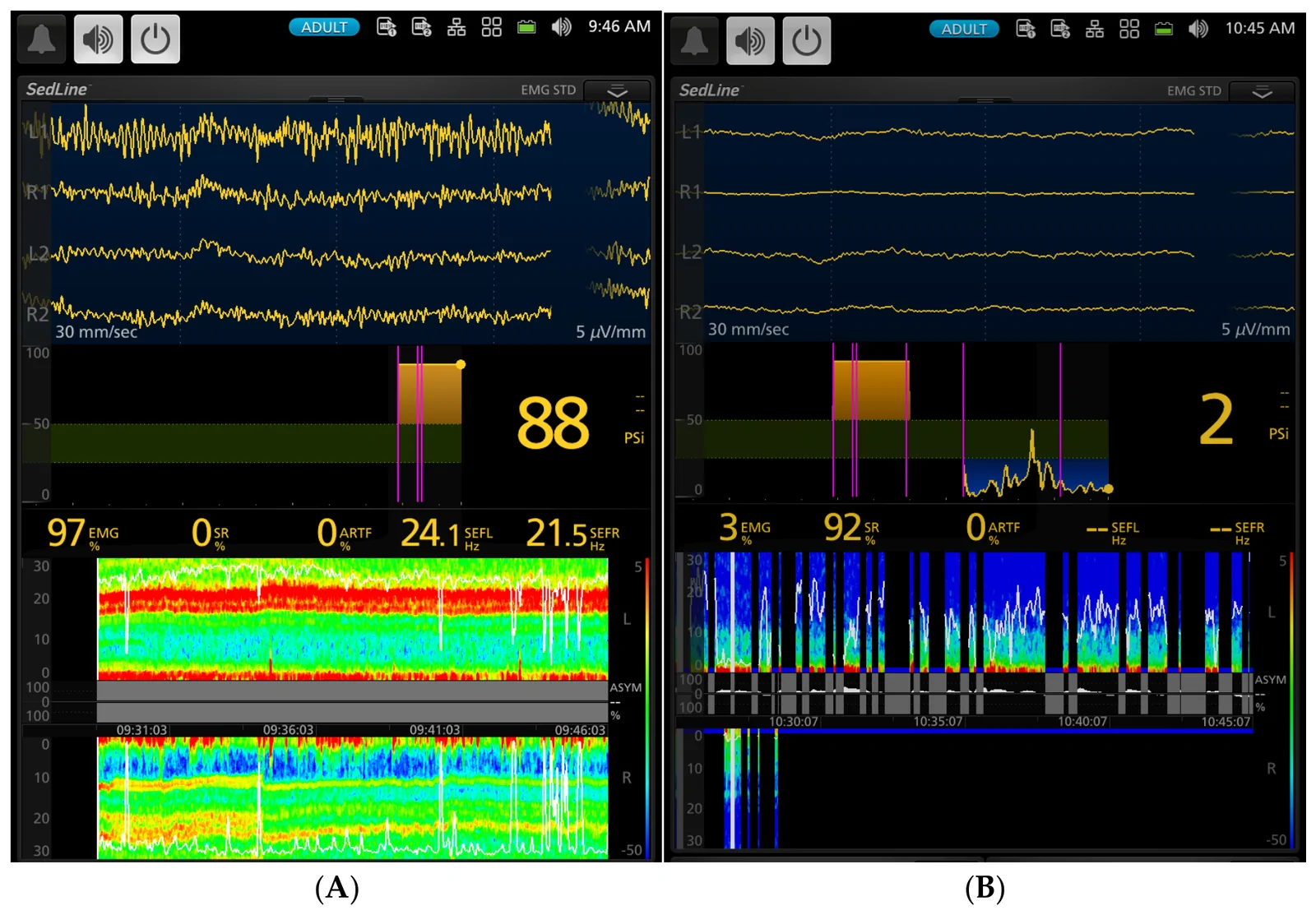
Electroencephalogram (EEG) and Spectrogram Progression in a Fentanyl Infusion (FC) Treated Dog. (**A**) T2 (Induction and Surgical Preparation): This image illustrates a typical response to a multimodal anesthetic protocol that includes ketamine. The dog demonstrates a ketamine-dominant brain state soon after induction and during the early isoflurane maintenance stage (Time stamp 9:46 AM). The three vertical pink lines on the display (from left to right) represent the exact timing of induction, endotracheal intubation, and the initiation of isoflurane anesthesia. The raw EEG displays high-frequency, low-amplitude gamma waveforms. This active cortical state is reflected in a Patient State Index (PSI) of 88 and Spectral Edge Frequencies (SEF-L: 24.1 Hz; SEF-R: 21.5 Hz), indicating that 95% of the spectral power lies below the upper gamma range. The density spectral array (DSA) spectrogram highlights prominent high-power (red) banding between 20 and 30 Hz across both hemispheres. Additionally, strong electromyographic (EMG) activity (97%) is present at the frontal facial muscles, likely induced by ketamine, with a complete absence of burst suppression (SR: 0%). This figure illustrates a ketamine dominant cortical state during the early phase of anesthesia, prior to the onset of global cortical depression. (**B**) T4 (Early Maintenance): By 10:45 AM, the initial ketamine-dominant state has completely subsided following drug metabolism, replaced by profound, multimodal cortical depression. In sharp contrast to Panel A, the raw EEG (upper panel) displays a nearly isoelectric (flat) trace. The PSI has plummeted from 88 to a critically low 2, and the suppression ratio (SR) has surged from 0% to 92%. Because of this severe lack of brain electrical activity, the SEF-L and SEF-R values cannot be calculated by the monitor and do not appear. Excellent muscle relaxation is also demonstrated by a reduction in EMG from 97% to 3%. The density spectral array (DSA) reveals an asymmetric suppression pattern: the right hemisphere is more profoundly affected, displaying large, solid black blocks with blue lower edges consistent with prolonged burst suppression, whereas the left hemisphere shows sparse brain activity interrupted by frequent burst-suppression patterns (vertical black bars). Furthermore, the PSI trendline (middle panel) visually captures this entire trajectory. It drops precipitously from the initial highly active state (bright yellow block on the far left), falls completely out of the optimal target range (green zone with PSI 25–50 in the PSI trend panel), and settles into a deep blue color on the far right, reflecting a profoundly depressed brain state driven by the combination of the multimodal anesthesia protocol with isoflurane and the fentanyl CRI.

**Figure 7:**
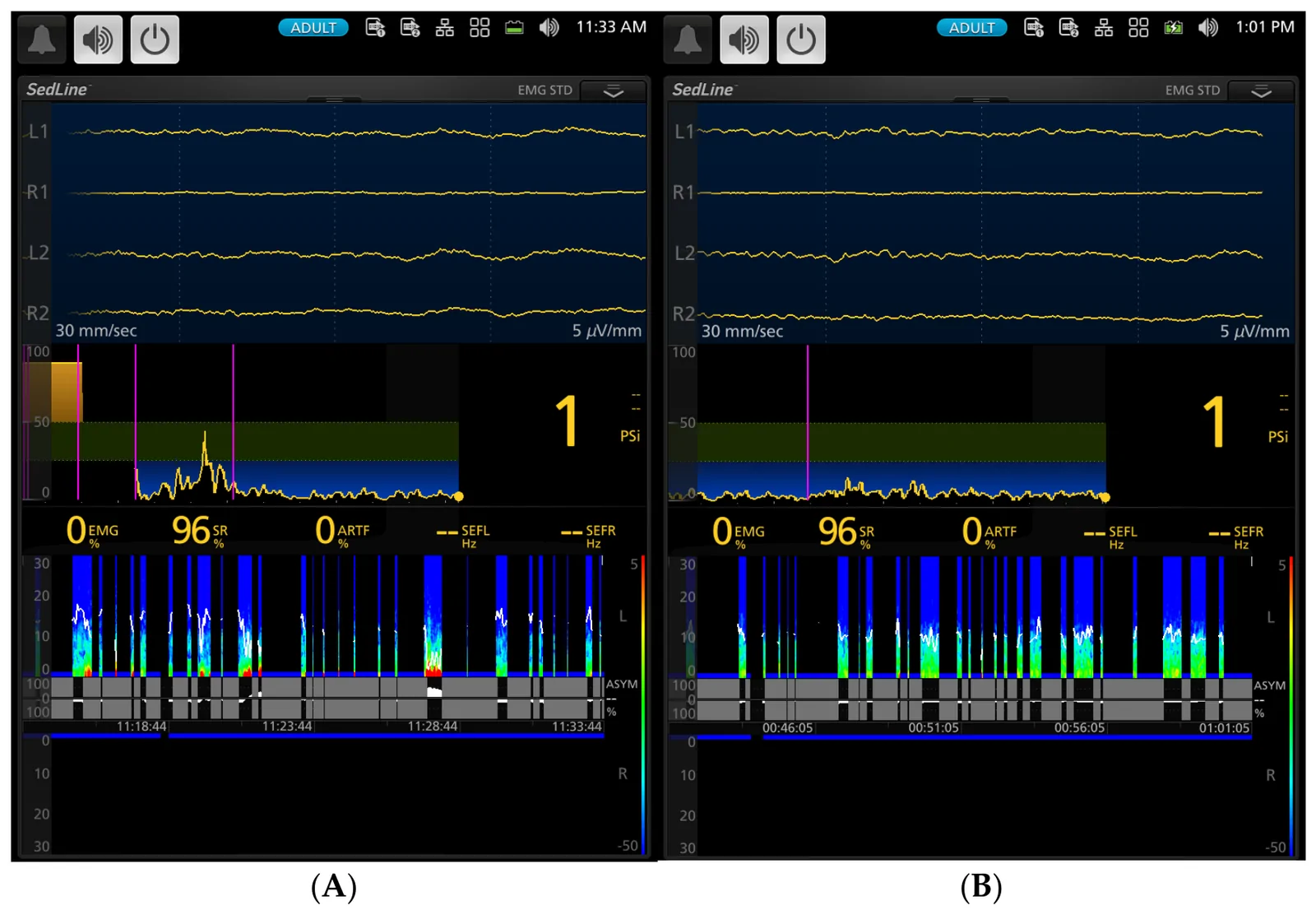
(**A**,**B**) T4 and T8 (Sustained Intraoperative Maintenance). Because these two time points (11:33 AM and 1:01 PM, respectively) exhibit nearly identical neurophysiological profiles, they are described collectively to illustrate the sustained, profound cortical depression maintained throughout the surgical procedure. In both panels, the raw EEG traces remain nearly isoelectric. The PSI has reached a nadir of 1, which the PSI trendline (middle panel) shows falling far below the optimal target range (the green zone, PSI 25–50) and remaining continuously within the deep blue zone. The suppression ratio (SR) is critically high at 96%, and complete muscle relaxation is evident with an EMG of 0%. Due to this severe lack of electrical activity, no SEF values can be calculated. The density spectral array (DSA) and asymmetry graphs consistently demonstrate that the right hemisphere remains more profoundly depressed than the left; it appears as a persistent black block in the lower DSA panel (right hemisphere) and shows some brain activity, with frequent black bars, in the upper panel (left hemisphere).

**Figure 8:**
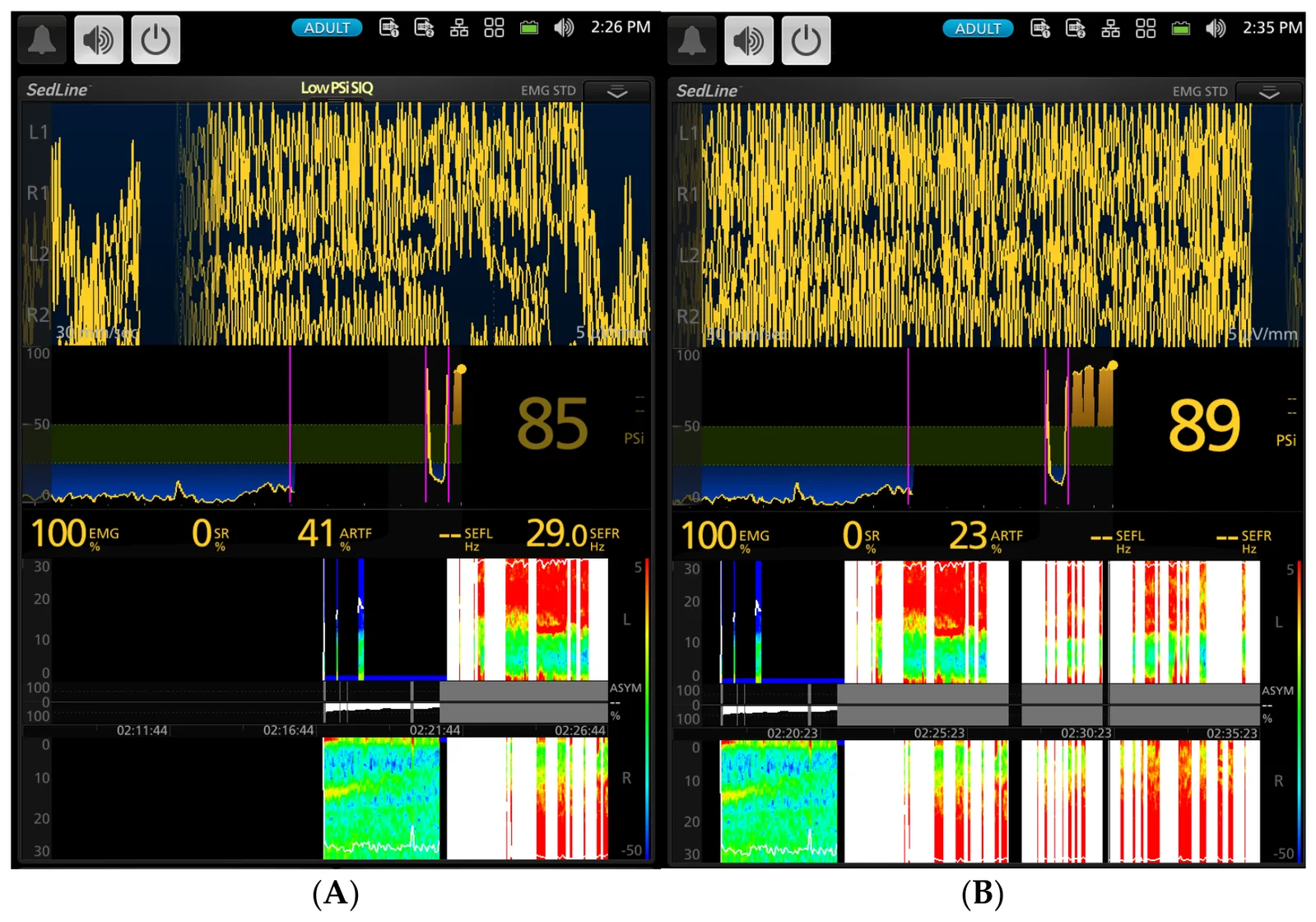
(**A**,**B**) T12 (Immediate Recovery). Because the two images represent a continuous emergence from 2:26 PM to 2:35 PM during the immediate recovery phase in T12, they are described collectively. Following the termination of isoflurane and the fentanyl CRI, the brain rapidly emerges from the profoundly depressed state observed throughout surgical maintenance. The Patient State Index (PSI) trendline (middle panel) illustrates a sharp, near-vertical ascent from the deep blue zone back into the highly active yellow range, with current PSI values rebounding to 85 and 89. The raw EEG traces (upper panels) transition abruptly from nearly isoelectric lines to active, high frequencies. This activity is strongly influenced by the return of muscle tone and physical movement, as corroborated by the electromyography (EMG) signal spiking to 100%. Patient movement during this awakening phase introduces significant interference, as evidenced by elevated artifact levels (ARTF 41% and 23%) and stark white vertical gaps across the density spectral array (DSA). The suppression ratio (SR) has returned to 0%. Together, these indicators reflect a rapid return of cortical and muscular activity during early emergence when the brain regains consciousness after a prolonged depressive brain state.

To visually illustrate these neurophysiological transitions, Figure 6, Figure 7 and Figure 8 provide representative SedLine monitor screenshots capturing distinct brain states in an FC-treated dog. This specific patient (Dog ID 5) was selected as a representative case because its documented clinical emergence directly mirrored the profound neurophysiological shifts captured by EEG monitoring. Clinically, this dog experienced a rough recovery characterized by vocalizations, necessitating rescue sedation with hydromorphone and dexmedetomidine. The provided image sequence tracks the progression from an initial ketamine-dominant phase following propofol-ketamine induction, through deep intraoperative cortical depression characterized by high burst suppression ratios during isoflurane and fentanyl maintenance, and ultimately to the abrupt cortical awakening that preceded this rough early recovery.

## 4. Discussion

The most significant finding of this study is that dogs frequently exhibited profound intraoperative cortical suppression despite maintaining clinically acceptable cardiovascular variables, revealing a substantial gap in routine anesthetic assessment.

The monitoring paradox arose because autonomic variables remained stable even when cortical activity showed substantial suppression. This monitoring paradox likely reflects an evolutionary defense mechanism, analogous to observations in humans, where subcortical autonomic centers in the brainstem remain highly resilient to anesthetic-induced depression to preserve vital cardiorespiratory functions, even while higher cortical networks succumb to profound suppression. The groups displayed similar HR, Et-Iso, SBP, MBP, DBP, and PTA values, indicating preserved cardiovascular homeostasis supported by brainstem autonomic centers [1,3,7,14,15,16,17]. In contrast, the processed EEG revealed deep cortical depression, with the FC group demonstrating the greatest susceptibility. This pattern became most apparent at the onset of surgical stimulation. At T3, PSI decreased from T1 by 53.8 in FC dogs, 51.9 in ME dogs, and 23.2 in RB dogs. SR values increased by 31.8%, 22.8%, and 1.6% in the FC, ME, and RB groups, respectively. These findings show that the transition from pre-incisional conditions to early surgical stimulation produced measurable shifts in cortical state across all groups, and that the FC protocol resulted in the deepest and most consistent cortical depression despite similar autonomic profiles.

In this study, dogs receiving fentanyl CRI demonstrated substantially deeper cortical depression than those receiving epidural morphine, a difference best explained by pharmacokinetic and neuroanatomical factors rather than μ-receptor density alone. Epidural morphine is hydrophilic, enters the central nervous system slowly, and is subject to P-glycoprotein efflux at the blood–brain barrier. As a result, its distribution remains predominantly within the spinal cord, limiting its supraspinal hypnotic impact despite prolonged neuraxial residence [32]. In contrast, fentanyl is highly lipophilic, rapidly enters the central compartment, and achieves widespread supraspinal μ-receptor occupancy. This produces profound cortical suppression and pronounced MAC sparing through synergistic interactions with isoflurane and propofol [33,34,35,36,37]. Thus, the difference in cortical depression between the two opioids reflects the quality and distribution of receptors occupied (brain vs. spinal cord), rather than the absolute number of receptors engaged.

Comparative veterinary studies confirm that systemic fentanyl infusions exert stronger CNS-level effects than epidural morphine, with fentanyl-based protocols producing deeper hypnotic states and more pronounced systemic opioid effects, whereas epidural morphine’s actions remain segmental and spinal [32,33]. Prolonged fentanyl infusions significantly reduce isoflurane MAC and deepen CNS depression in dogs, reinforcing the susceptibility of cortical networks to systemic μ-agonism [33]. Furthermore, EEG-based depth studies demonstrate that cortical suppression can be profound even when autonomic responses remain unchanged, supporting the concept that opioid–volatile synergy can decouple cortical and cardiovascular states [34].

Although this study primarily explored the feasibility of EEG monitoring in an orthopedic dog population, the findings also highlight an important mechanistic insight: both fentanyl CRI and epidural morphine produced episodes of marked frontal cortical depression, reflected by low PSI values and high SR, demonstrating that both opioids are capable of driving the cortex into a profoundly suppressed electrical state under certain anesthetic conditions. However, translational EEG and PK/PD literature show that similar EEG signatures can arise from fundamentally different network mechanisms. In humans, increasing fentanyl concentrations produce progressive slowing of cortical activity and reductions in β-band power [35,36], and at higher effect-site concentrations, fentanyl induces a frontal θ (4–8 Hz) biomarker reflecting deep supraspinal μ-agonism and thalamocortical suppression [37]. Pharmacokinetic–pharmacodynamic modeling further demonstrates that fentanyl equilibrates rapidly with the brain, whereas morphine exhibits complex biophase distribution and limited supraspinal penetration due to hydrophilicity and P-glycoprotein transport [38]. In pediatric patients, morphine produces distinct cortical signatures—reductions in β1 and β2 power and decreased frontal–occipital coherence—that correlate strongly with respiratory rate depression, underscoring a tight coupling between cortical arousal and ventilatory control [39]. Collectively, these data support the interpretation that, although both fentanyl and morphine can converge on similar EEG manifestations such as low PSI, high SR, and burst suppression, fentanyl CRI produces greater cortical depression because it delivers higher and more homogeneous brain μ-receptor activation, whereas epidural morphine’s spinal-dominant distribution limits its hypnotic impact despite producing similar EEG endpoints under certain anesthetic conditions.

It is worth noting that the EEG findings reported by Thomas et al. (2025) [34] in dogs anesthetized with sevoflurane closely parallel the cortical responses observed in our orthopedic population, despite important differences in anesthetic protocol and fentanyl dosing. In their study, dogs receiving a fentanyl bolus (2 µg/kg IV) followed by a continuous infusion of 0.2 µg/kg/min—a rate approximately 2.4-fold higher than the fentanyl CRI used in our study (5 µg/kg/h = 0.083 µg/kg/min)—demonstrated profound cortical suppression, with PSI values falling to 2, indicating near-isoelectric cortical activity. Burst suppression was consistently observed, reflected by high SR values, and the effective sevoflurane concentration for inducing burst suppression was 3.15%. Importantly, electrical stimulation increased mean arterial pressure without corresponding changes in PSI, and heart rate remained stable, demonstrating that brainstem autonomic reflexes remained functional even when cortical networks were profoundly depressed.

Our study, conducted in an orthopedic clinical setting, employed a lower fentanyl infusion rate and incorporated multimodal premedication and induction typical of orthopedic anesthesia. Despite this lower fentanyl exposure, our dogs exhibited the same EEG phenomena described by Thomas et al.—marked reductions in PSI, elevations in SR, and episodes of burst suppression—underscoring the sensitivity of canine cortical networks to opioid–volatile synergy. The similarity between studies, despite differences in fentanyl dose, anesthetic depth, and surgical context, reinforces our proposed mechanistic interpretation: the cortex and brainstem respond differently under opioid–volatile anesthesia, with cortical networks demonstrating profound susceptibility to suppression while brainstem autonomic centers maintain functional responsiveness. This cortical–brainstem dissociation explains why autonomic variables (MAP, HR) remained stable or reactive in both studies even when EEG indices indicated deep cortical depression, and it provides a coherent physiological framework for interpreting the monitoring paradox observed in our orthopedic cohort.

General anesthetics depress subcortical arousal systems while disrupting cortical networks responsible for the content of consciousness, particularly frontoparietal circuits involved in large scale integrative processing [3,6,7]. They also impair reciprocal communication between the cortex and thalamus, particularly via matrix cell pathways that coordinate widespread cortical information flow [6,7,20]. Mathematical and computational models show that anesthetics reduce ascending arousal from the brainstem and weaken synaptic connectivity within both cortex and thalamus [3,6,8,19,20,21]. These combined effects produce the EEG slowing and burst suppression patterns observed in this study [5,7,21,22,28,29]. Because anesthetics preferentially disrupt horizontal cortical communication while leaving ascending nociceptive pathways relatively intact [3,6,7], surgical stimuli can still activate subcortical networks. This activation produces catecholamine mediated sympathetic responses that maintain autonomic vital signs even when cortical networks are fragmented [6,7].

PTA monitoring provided additional clarity by continuously tracking heart rate variability, which serves as a surrogate for parasympathetic tone [15,16]. The observed preservation of autonomic balance during surgical stimulation, as indicated by PTA fluctuations that did not differ among treatment groups, biologically reflects the successful blockade of ascending nociceptive pathways before they trigger massive sympathetic outflow. While EEG recordings showed profound cortical suppression, this preservation of autonomic balance underscores the functional separation between brainstem-driven physiological responses and higher cortical integrity. These findings support the notion that subcortical nociceptive processing and cardiovascular homeostasis persist independently of cortical state in this clinical model. The continuous EEG progression of individual dogs further illustrates this dissociation. A representative FC dog (Dog ID 5) demonstrated an active ketamine dominant cortical state following induction and early isoflurane maintenance (Figure 6A), characterized by high frequency, low amplitude waveforms, consistent with previous work [29]. Pharmacologically, ketamine acts as an NMDA receptor antagonist, uncoupling the thalamocortical axis and resulting in paradoxical cortical excitation and high-frequency gamma activity even during unconsciousness [3,30]. This pronounced cortical activity was not detectable clinically; the dog appeared relaxed and stable despite substantial EMG activity. As surgery progressed and the fentanyl infusion reached full effect (Figure 6B and Figure 7B), the EEG transitioned into profound cortical depression, culminating in a nearly isoelectric state with a PSI value of 1 and a burst suppression ratio approaching 100%.

To systematically evaluate cortical and autonomic dynamics, we applied the Double-Low and Triple-Low concepts adapted from human perioperative literature [9,10,11,12,18,27]. Low cortical activity was defined as PSI ≤ 25; substantial suppression as SR ≥ 20%; low inhalant requirement as Et-Iso < 1.02%; and hypotension as MBP ≤ 60 mmHg. These thresholds were selected because human studies consistently show that concurrent low cortical activity, low volatile anesthetic requirement, and hypotension identify periods in which cortical integrity, perfusion, and anesthetic depth are simultaneously compromised, a state that is strongly associated with poor recovery outcomes such as postoperative delirium, prolonged hospitalization, and increased mortality [9,10,11,12,18,27]. In the current study, a PSI ≤ 25 falls below the standard surgical range of 25–50. This target range was recently utilized in human clinical trials specifically to guide volatile anesthetic administration and avoid burst suppression [17,40]. Furthermore, a PSI ≤ 25 corresponds to markedly reduced cortical information processing [5,6,7]. SR ≥ 20% identifies periods of substantial suppression, paralleling human definitions of deep cortical depression [5,6,7]. Et-Iso < 1.02% corresponds to approximately 0.8 MAC in dogs [23,24,25,26]. MBP ≤ 60 mmHg reflects a clinically meaningful reduction in perfusion pressure [1,3]. Together, these criteria provide a species-modified, physiologically grounded adaptation of the human Double-Low and Triple-Low constructs and allow structured evaluation of cortical state, autonomic stability, and anesthetic depth in dogs [3,5,6,7,9,10,11,12,17,18,23,24,25,26,27].

This framework clarifies the severe cortical depression observed in several dogs. In the example, the FC-treated dog (Figure 7A,B) showed nearly isoelectric EEG waveforms and high burst suppression while blood pressure, heart rate, and Et-Iso concentrations remained clinically stable. While this dog provides a clear visual example, similar Double-Low states occurred across all treatment groups, indicating that these profound depressions reflected the combined influence of the multimodal anesthetic protocol, individual anesthetic sensitivity, and the specific analgesic technique used. Specifically, this phenomenon relates directly to the divergent mechanisms of action of the analgesic treatments: whereas systemic opioids (FC) act centrally to depress broad cortical and subcortical neural networks, regional blocks (RB) intercept nociceptive transmission peripherally, thereby sparing the cortex from additional direct pharmacological depression. Importantly, this level of cortical suppression was entirely undetectable from outward clinical signs; the dogs appeared appropriately anesthetized and stable despite marked EEG depression. Autonomic variables also showed treatment-specific temporal patterns, but these patterns did not align with the depth or direction of EEG changes. Whereas PSI and SR reflected differences in cortical suppression among protocols, SBP and MBP appeared to reflect cardiovascular compensation and responses to surgical stimulation. Thus, clinically acceptable arterial pressures should not be interpreted as evidence that cortical activity is similarly preserved. These observations demonstrate that multimodal anesthesia can produce deep alterations in brain state that remain invisible when relying solely on hemodynamic stability, and that EEG monitoring is necessary to identify these episodes. This recognition naturally leads to the question of how different analgesic techniques influence cortical activity when layered onto the same anesthetic background.

The multimodal anesthetic protocol allowed us to examine how three analgesic techniques modulated cortical state within the shared background of hydromorphone, dexmedetomidine, ketamine, propofol, and isoflurane. Because FC, ME, and RB intercept nociception at different anatomical levels, their cortical effects diverged substantially [3,5,6,7,22]. FC produced the most pronounced cortical depression, consistent with the expected supraspinal effects of systemic opioid administration. RB and ME produced less marked or less consistent cortical suppression, likely reflecting differences in how peripheral, neuraxial, and systemic analgesic techniques modulate nociceptive input and cortical arousal [1,5,7]. These findings support the interpretation that cortical EEG monitoring can reveal treatment-related differences in brain state that are not apparent from conventional autonomic variables alone.

Double-Low analysis further highlighted these discrepancies and provided a mechanism to identify individual patient sensitivity to the multimodal anesthetic protocol [9,10,11,27]. Across all dogs, 71 Double-Low events were identified. FC dogs exhibited the highest overall incidence (39 total events) with high-frequency clustering across the cohort (individual dog event counts of 9, 7, 7, 7, 5, 4, and 0). This uniform distribution supports the conclusion that continuous systemic opioid administration drives consistent, profound cortical depression across a population. Conversely, tracking Double-Low frequencies revealed significant inter-individual variability in the other groups, particularly the RB group (20 total events). Although regional blocks spare the cortex from the direct central depression of systemic opioids, two dogs in the RB group demonstrated notable sensitivity to the anesthetic protocol, exhibiting frequent Double-Low states (individual event counts of 7, 6, 3, 2, 2, 0, and 0). This high variance illustrates that even without systemic opioids, the synergistic interaction between isoflurane and the baseline multimodal anesthetics can still drive low PSI and high SR in susceptible individuals. ME dogs showed a similarly sparse and infrequent Double-Low distribution (12 total events; individual counts of 4, 4, 3, 1, 0, 0, and 0) [27]. These distributions demonstrate that using a Double-Low index is highly effective for identifying individual sensitivity to synergistic anesthetic combinations that may otherwise be masked by stable cardiovascular variables.

Emergence times were comparable across groups, indicating that the depth of intraoperative cortical depression did not prolong extubation. However, EEG profiles at the end of surgery demonstrated substantial differences in cortical state. FC dogs had the most depressed EEG profile at the end of surgery, with very low PSI and high SR. ME dogs appeared less suppressed, whereas RB dogs remained suppressed but with a different profile. By T11, PSI and SR values had become more similar across groups, suggesting that cortical recovery began rapidly after inhalant discontinuation. The key point is not delayed emergence time, but the abrupt transition from markedly suppressed cortical activity to wakefulness in dogs with deeper intraoperative depression, particularly in the FC group [5,6,7,21,22,28,29].

Recovery quality did not differ significantly among groups, but descriptive patterns were clinically meaningful. Rough or delirious recoveries occurred in 3 of 7 FC dogs, 4 of 7 ME dogs, and 2 of 7 RB dogs. Rescue sedation or analgesia was required in 3 FC dogs, 4 ME dogs, and 2 RB dogs. These observations parallel human findings in which intraoperative burst suppression is associated with postoperative delirium and impaired cognitive recovery [5,6,7,18,21,41]. Dogs across all groups transitioned from deep cortical suppression to wakefulness while still under the influence of the multimodal protocol. This rapid shift likely created a neurophysiologic disequilibrium that predisposed these dogs to agitation, disorientation, and impaired sensory integration during emergence [21,41]. This phenomenon is illustrated in Dog ID 5 (Figure 8A,B), where a rapid ascent of PSI from deep suppression to high activity corresponded with a rough recovery requiring rescue sedation.

Postoperative pain scores and 24 h rescue analgesia requirements further contextualize these findings. Peak Glasgow pain scores were low across groups, yet rescue analgesia was required in 3 FC and 2 ME dogs, whereas no RB dogs reached the intervention threshold. The absence of rescue analgesia in RB dogs suggests that more stable cortical activity during surgery may support smoother emergence and more consistent postoperative comfort [30]. Together, these results indicate that the degree of intraoperative cortical suppression, rather than emergence time or autonomic stability, may be a key contributor to the quality of recovery and postoperative neurobehavioral outcomes [5,6,7,18,21,41].

This study has several limitations. Respiratory assessment was limited to Et-CO2 and Et-Iso because all dogs were mechanically ventilated to optimize PTA acquisition. This prevented the evaluation of spontaneous ventilation and masked the respiratory depressive effects of opioids, which are commonly used as clinical indicators of anesthetic depth [3,22]. Although an a priori power analysis was conducted to ensure adequate power for the primary outcomes, the cohort size of 7 dogs per group remains relatively small. This limited sample size, coupled with inherent inter-individual variability in anesthetic sensitivity, likely contributed to a lack of statistical significance in some between-group comparisons. Nevertheless, this sample size successfully captured robust, highly significant temporal shifts in cortical state across the entire cohort, validating the primary premise that these monitors reliably detect clinically meaningful neurophysiological changes. Blinding of anesthesiologists was not feasible due to practical constraints; however, potential observer bias was minimized as the primary outcome measures (PSI, SR, and PTA) were derived directly from automated, objective algorithms. Additionally, because this study used proprietary algorithms specific to the SedLine and PTA monitors, the absolute values and Double-Low thresholds established here may not be directly comparable to those of other depth-of-anesthesia or nociception monitors. Furthermore, while the numerical thresholds defining the canine Double-Low state were grounded in physiological principles and human literature, they have not yet been prospectively validated against long-term postoperative morbidity or mortality outcomes in veterinary medicine. These species-adapted criteria provide a necessary foundational framework for future large-scale investigations into canine neurobehavioral outcomes. Finally, the cohort consisted of large-breed, systemically healthy dogs undergoing TPLO procedures, representing a specifically orthopedic and somatic pain model. These findings may not generalize to geriatric, pediatric, frail, or critically ill patients, nor to patients undergoing procedures involving severe visceral nociception.

## 5. Conclusions

In conclusion, evaluating the cardiorespiratory and cortical responses using frontal EEG and PTA demonstrates that standard autonomic monitoring frequently masks severe intraoperative cortical depression, creating a clinical blind spot that results in Double-Low states. The three evaluated analgesic techniques induced distinct EEG characteristics due to their differing pharmacokinetic properties and neuroanatomical distributions. Notably, systemic fentanyl and epidural morphine exhibit different profiles in inducing EEG patterns of low PSI and high suppression ratios, with lipophilic fentanyl driving more profound and consistent supraspinal depression than spinal-dominant morphine. Furthermore, even in the regional block group among individual dogs with higher sensitivity to anesthetics, the synergistic interaction between inhalants and the underlying multimodal protocol—containing opioids and other anesthetics—drives profound cortical depression while simultaneously maintaining brainstem-driven cardiovascular responses, thereby masking the true depth of cortical suppression in dogs undergoing TPLO procedures. Because all evaluated analgesic protocols effectively decouple brainstem-driven hemodynamic stability from cortical consciousness, rapid emergence from these deeply suppressed cortical states predisposes dogs to dysphoric recoveries. Therefore, integrating EEG and PTA monitoring alongside conventional cardiorespiratory assessment offers a valuable approach to evaluate true brain state, optimize neurophysiological stability, and anticipate challenging postoperative recoveries.

## Figures and Tables

**Table 1:** Patient Demographics, Body Weight, Anesthetic and Surgical Durations, and Procedural Distribution Across Treatment Groups. Data presented as (Mean ± SD or *n*), MN: male neuter, FS: female spayed. MPL = Medial Patellar Luxation, and TPLO = Tibial Plateau Leveling Osteotomy.

Parameter	FC Group (*n* = 7)	ME Group (*n* = 7)	RB Group (*n* = 7)
**Sex (Male/Female)**	2 MN/5 FS	5 MN/2 FS	1 MN/6 FS
**Age (years)**	7.3 ± 3.2	5.4 ± 2.2	5.9 ± 2.9
**Body Weight (kg)**	40.7 ±10.1	31.6 ± 7.2	40.8 ± 8.8
**Surgical Duration (min)**	124.5 ± 35.8	108.2 ± 28.4	114.6 ± 31.2
**Anesthesia Duration (min)**	274.0 ± 52.8	235.7 ± 36.6	231.3 ± 49.3
**Procedures (*n*)**			
**Arthroscopy + TPLO**	5	4	3
**TPLO only**	1	2	4
**Stifle Osteotomy/MPL**	1	1	0

**Table 2:** Incidence and Distribution of Double-Low States Across Analgesic Treatment Groups in Dogs Undergoing Tibial Plateau Leveling Osteotomy. Summary of Double-Low state incidence, distribution, and statistical comparisons across the three analgesic protocols. The table reports the number of dogs affected, total Double-Low time points, individual event counts, and overall distribution patterns for each treatment group (FC, ME, RB). All Double-Low events consisted of cortical suppression (Low Brain State) paired with low inhalant requirements; no instances of hypotension combined with low inhalant requirements were observed. Statistical comparisons among groups were performed using mixed-effects logistic regression.

Treatment Group	Dogs with Double-Lows (*n*/7)	Total Double-Lows Time Points	Individual Dog Event Counts	Distribution Pattern	Double-Low Type
**FC**	6/7 (86%)	39	9, 7, 7, 7, 5, 4, 0	High-frequency clustering across most dogs	All Double-Lows were due to cortical suppression (Low Brain State) paired with Low Inhalant Requirement; no hypotension + low inhalant combination
**ME**	4/7 (57%)	12	4, 4, 3, 1, 0, 0, 0	Sparse, low-frequency distribution	Same as FC group
**RB**	5/7 (71%)	20	7, 6, 3, 2, 2, 0, 0	High variance: two dogs high, others low/zero	Same as FC group

## Data Availability

The original contributions presented in this study are included in the article. Further inquiries can be directed to the corresponding author.

## Abbreviations

The following abbreviations are used in this manuscript:

EEG	Electroencephalography
PTA	Parasympathetic tone activity
BIS	Bispectral Index
PSI	Patient State Index
TPLO	Tibial plateau leveling osteotomy
FC	Fentanyl constant rate infusion
ME	Morphine epidural
RB	Saphenous and sciatic regional nerve block
Et-CO_2_	End tidal carbon dioxide
Et-Iso	End tidal isoflurane
HR	Heart rate
SBP	Systolic blood pressure
MBP	Mean blood pressure
DBP	Diastolic blood pressure
SR	Suppression Ratio
SEF-L	95% Left Spectral Edge Frequency
SEF-R	95% Right Spectral Edge Frequency
DSA	Density Spectral Array
mPTA	Median PTA
CMPS-SF	Glasgow Composite Measure Pain Scale-Short Form
CIs	Confidence intervals
